# Trends in Use of Prescription Skin Lightening Creams

**DOI:** 10.3390/ijerph18115650

**Published:** 2021-05-25

**Authors:** Dana S. Saade, Mayra B. C. Maymone, Henriette De La Garza, Eric A. Secemsky, Kevin F. Kennedy, Neelam A. Vashi

**Affiliations:** 1 Department of Dermatology, Boston University School of Medicine, Boston, MA 02118, USA; ds45@aub.edu.lb (D.S.S.); mayrabcm@gmail.com (M.B.C.M.); hadelaga@bu.edu (H.D.L.G.); 2 Department of Medicine, Division of Cardiology, Beth Israel Deaconess Medical Center, Boston, MA 02215, USA; ericsecemsky@gmail.com; 3 Saint-Luke’s Mid America Heart Institute and University of Missouri-Kansas City School of Medicine, Kansas City, MO 64111, USA; kfkennedy@saint-lukes.org

**Keywords:** lightening cream, hyperpigmentation, skin of color, quality of life, melasma

## Abstract

The desire for an even skin tone pervades all cultures and regions of the world. Uniform skin color is considered a sign of beauty and youth. Pigmentation abnormalities can arise idiopathically with genetic predetermination, with injury and environmental exposures, and with advancing age, and can, therefore, be distressing to patients, leading them to seek a variety of treatments with professional assistance. In this short report, we describe the trends in the use of prescription lightening creams, particularly in patients with darker skin types residing in the US. Amongst 404 participants, skin hyperpigmentation had a moderate effect on patients’ quality of life, and the most common diagnosis associated with the use of a prescription product was melasma (60.8%). The most common agent prescribed was hydroquinone (62.9%), followed by triple combination cream (31.4%). It is the dermatologist’s duty to gauge the effect of the pigmentation disease on patients’ life in order to counsel, tailor, and decide on the most appropriate treatment option.

## 1. Introduction

The desire for an even skin tone pervades all cultures and regions of the world. Uniform skin color is considered a sign of beauty and youth, therefore, abnormalities in pigmentation can be distressing to patients, leading them to seek a variety of treatments with professional assistance. The pigmentation of the skin depends on several factors including the amount of melanin, degree of skin vascularity, presence of carotene, and thickness of the stratum corneum [1]. Skin hyperpigmentation usually results from an increased number or activity of melanocytes [1]. The most common abnormal pigmentation results from photoinduced damage to the skin in the form of freckles, lentigines, and maturational hyperpigmentation [2]. In addition to these, other common types of pigmentation disorders include melasma, periorbital melanosis, and post-inflammatory hyperpigmentation (PIH) [2]. Multiple factors including ultraviolet (UV) light and a variety of hormones are known to affect pigment cell function and proliferation and create alterations in pigmentation [3]. Skin lightening, also known as skin bleaching, refers to the use of products to lighten dark areas of the skin or achieve an overall lighter complexion. These products include creams, soaps, and pills, as well as professional treatments such as chemical peels and laser therapy. Skin bleaching reduces the concentration or production of melanin in the skin. Skin color varies depending on the quantity and distribution of melanin-producing melanocytes [4]. Since darker-skinned individuals have more melanin, hyperpigmentation disorders and the use skin depigmentation agents are common among people with skin of color both in the US and worldwide [5]. It is of utmost importance that dermatologists are informed about the products available for cosmetic skin lightening as well as their proper use and possible side effects. Hereunder, we describe United States (US) prescription lightening product trends and its association with patients’ quality of life in those with disorders of hyperpigmentation.

## 2. Methods

In this study, approved by the Boston University Institutional Review Board, 404 consecutive patients ≥18 years of age completed a questionnaire in English, Spanish, or Portuguese. All patients presenting with a complaint of hyperpigmentation were asked to participate, with right to refuse. Questions were related to demographics, location of skin darkening, etiology, product prescribed, and quality of life (QoL) as measured by the validated Dermatology Life Quality Index (DLQI) [6]. Fitzpatrick skin type and diagnosis were determined by a board-certified dermatologist.

**Figure 1:**
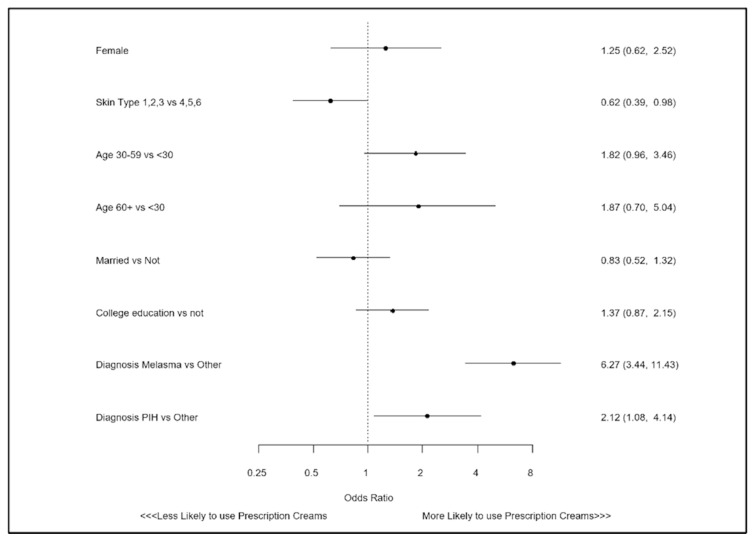
Factors associated with use of a prescription lightening creams.

Differences in patient characteristics were compared between prescription lightening cream users and non-users (Table 1). For continuous variables, data are shown as means with standard deviations, and for categorical variables as counts and rates. To compare continuous data, students t-tests were used and for categorical data, chi square tests. To determine independent predictors of prescription product use, a logistic regression model was created. Candidate variables were selected based on prior literature and clinical plausibility, and included sex, age, skin type, marital status, education, and hyperpigmentation disorder (Figure 1). Data analysis was performed with Statistical Analysis System (SAS) version 9.4 (Cary, NC, USA), with a *p*-value < 0.05 considered to be statistically significant. 

## 3. Results

A total of 404 patients with hyperpigmentation disorders were analyzed. Among these patients, the average age was 41.3 +/− 12.3, and the majority were immigrants to the USA (70.3%), female (88.6%), and had skin types IV–VI (64.4%). Of all patients, 46% were currently using a prescription lightening cream. 

The most common hyperpigmentation skin disorder amongst patients using prescription lightening agents was melasma (60.8%), a common and acquired skin disease characterized by hyperpigmented macules and patches on the face [2]. The frequency of disorders among non-prescription users varied between melasma (28.6%), PIH (37.6%), and other disorders (33.8%). When patients were queried whether they were familiar with their hyperpigmentation diagnosis, the majority (>64% in both groups) were unsure. However, in the prescription user group, a larger number knew what melasma was (35.3%) as compared to those not using any prescription creams (18.9%) (*p* < 0.001). Although 78.3% of patients using prescription lightening creams also used sunscreen regularly, exceeding those not using prescription creams (58.2%, *p* < 0.01), and 36.6% did not know that the sun was a possible cause or aggravating factor of their pigmentation.

Although most participants felt that the first person to contact regarding their pigmentation was a doctor, this was more apparent in those who were using prescription lightening creams (73.9%) versus non-prescription users (55.6%) (*p* < 0.001). In both groups, prescription users (95.7%) and non-prescription users (98.6%) believed that the best option to treat their hyperpigmentation was a with a dermatologist.

Regarding the content of the prescription creams, hydroquinone was the most commonly prescribed (62.9%), followed by triple combination cream (TCC—fluocinolone acetonide, hydroquinone, and tretinoin) (31.4%). Azelaic acid and topical steroids were also among the prescribed components, although less frequently. In terms of subjective efficiency, 69.8% felt that triple combination cream provided the best result, followed by 36.1% for hydroquinone. Azelaic acid and prescription steroids were felt to be effective in less than 20% of the responders. 

In regard to quality of life, the overall DLQI for patients with hyperpigmentation was 7.319 ± 5.21, which is consistent with a moderate impact based on prior literature [7]. DLQI scores were numerically higher among prescription users, but this did not reach statistical significance (7.59 vs. 7.09, *p* = 0.330). The most affected domains were embarrassment and level of self-consciousness regarding their condition and the effect it had on their social and leisure activities. 

In multivariate analysis, a diagnosis of melasma was independently associated with the greater use of prescription lightening creams (OR 6.27; 95% CI: 3.44,11.43; *p* < 0.001), as was PIH (OR 2.12; 95% CI: 1.08, 4.14; *p* = 0.029). In addition, there were lower odds of lightening agent use among those with lighter skin types (OR 0.62; 95% CI: 0.39, 0.98; *p* = 0.042).

## 4. Discussion

Hyperpigmentation disorders are one of the most common conditions seen by dermatologists, with approximately 24.7 million dermatology visits in the US made between 1994 and 2010 for their management [8]. Hyperpigmentation is a dermatologic condition that affects communities of color worldwide. Studies performed in the US found this condition in 65.3% of blacks, 52.7% of Hispanics, and 47.4% of Asians [9]. The treatment of hyperpigmentation requires reduction of melanin in the epidermis and dermis. There are various medical therapies that can be used, including depigmenting agents such as hydroquinone (HQ), topical corticosteroids, tretinoin, and other agents that act via tyrosinase-mediated pathways. Treatment modalities should be individualized depending on the patient’s skin type. There are several products containing HQ and other ingredients that are available OTC which can have adverse effects if not used correctly and followed by a dermatologist. Many OTC skin lightening products are not recommended for darker skin tones and could cause hyperpigmentation through different mechanisms. It is imperative that patients seek counsel from a dermatologist, since addressing hyperpigmentation disorders in patients of skin of color may be very challenging. As such, given the US’s changing demographics, it is critical for dermatologists to be knowledgeable about the medical impact as well as social and cultural implications of this practice [10]. Cosmetic skin lightening is a growing trend and continues to be popular despite well-documented adverse effects. The gold standard for hyperpigmentation therapy in the US is HQ. HQ is also the most extensively studied treatment for hyperpigmentation disorders. Studies have reported that lightening creams with HQ in concentrations of 2–5% are generally safe and efficacious [11]. However, patients commonly experience irritation, erythema, stinging, scaling, and occasional paradoxical postinflammatory hypermelanosis. With prolonged application, HQ can result in exogenous ochronosis characterized by progressive pigmentation of the area to which the agent is applied [11,12]. There have also been concerns of possible carcinogenicity. Renal adenoma has been found in genetically susceptible rats which are prone to chronic progressive nephropathy (CPN). However, it should be noted humans are not genetically prone to CPN and human metabolism of HQ yields less-toxic HQ conjugates than in rats [13]. Topical corticosteroids are commonly used skin lightening agents due to their potent bleaching action and anti-inflammatory activity. Nonetheless, they are also responsible for many of the side effects of skin bleaching [11]. Dermatologic side effects of topical corticosteroids include skin atrophy, striae, and acne, while systemic side effects, although exceedingly rare, include Cushing syndrome, hyperglycemia, and menstrual irregularities [14]. The most popular prescription retinoids used for direct improvement in skin pigmentation are tretinoin and tazarotene. Retinoids reduce the melanin content of the skin when used for a long period of time, helping to even out areas affected by hyperpigmentation [12]. A side effect of retinoids is an irritant dermatitis characterized by erythema, dryness, and scaling. As a result, preparations that combine tretinoin and hydroquinone also incorporate a topical corticosteroid to negate some of these known side effects [12]. TCC consists of HQ, a retinoid, and a fluorinated corticosteroid, and has become widely popular and regarded as a safe and effective treatment for several hyperpigmentation disorders [15]. It is important to note that the majority of lightening agent side effects, especially HQ, result from over-the-counter (OTC) formulations. Severe adverse reactions have been linked to extended use of HQ, moreover OTC concentrations of up to 8% obtained in other countries [16]. Easy accessibility with unsupervised usage of HQ can lead to overuse and increased risk for adverse events. Additionally, such OTC preparations often contain various additives that may contribute to the development of severe side effects such as exogenous ochronosis. Thus, when patients self-medicate, results are often unsatisfactory. This underlines the essential role of a dermatologist’s’ counsel and monitoring of a personalized treatment including the proper medication, correct usage, concentration and/or combination of agents to achieve safe and efficacious results.

In our descriptive study of a US-based population with darker skin types, patients on prescription creams were diagnosed with melasma in the majority of cases and most often used hydroquinone or TCC, with the latter having greater efficacy. Accompanying the prescribed treatment was a proper pairing with sunscreen. This is a positive finding, showing that management of such conditions is complete with sun protection. However, awareness regarding the sun contribution to their pigmentation can be improved; despite the use of sunscreen along with their prescribed creams, more than one third did not know that the sun was a possible cause or aggravating factor of their pigmentation. Furthermore, the cause of the hyperpigmentation was most often not clear to our responders. According to a survey of 1000 adults conducted by the Consumer Reports National Research Center, one-third of Americans do not use sunscreen, indicating that sun protection measures are not followed correctly, if at all [17]. Survey respondents said that they did not know that sunscreens with higher SPF afforded greater protection and that sunscreens past their expiration dates should not be used. Similarly, our responders were unaware that daily sunscreen application is vital as well as constant reapplication during the day. Education of causality and associated findings can be a timely process, especially within an immigrant population where language and customs may further complicate the situation; however, this is crucial and should be emphasized. Hyperpigmentation disorders such as melasma and PIH are photosensitive conditions which are exacerbated by exposure to UV light making sunscreen use an essential component of treatment. This is especially true in patients with skin of color who are less likely to use photoprotection due to the misconception that dark-skinned people do not need sunscreen [18]. According to Kang et al. a search of the National Ambulatory Medical Care Survey showed that photoprotection was the third most common treatment option in Caucasians, the sixth among African Americans and tenth among Asians [19]. This highlights the lack of awareness regarding the use of photoprotection in darker skin populations. The increased amount of melanin offers some protection from the sun by absorbing and distributing UV radiation. 

In fact, dark-skinned individuals have a natural skin protection factor (SPF) of up to 13, and filter twice as much UV radiation as lighter toned people. However, they are still susceptible to skin cancer and sun damage. In dark skinned individuals, the higher melanin content and more responsive melanosomes lead to a greater risk of hyperpigmentation, which is often more prominent and longer lasting when compared to lighter-skinned individuals. For this reason, dermatologists advise everyone, regardless of skin color, to use sunscreen with an SPF of at least 30 ideally with a physical sunblock such as titanium dioxide, zinc oxide, and iron oxide.

Although the weight that patients with hyperpigmentation disorders carry cannot be measured by the same physical endpoints as other chronic diseases, the psychosocial effects are undeniable. The negative impact on the QoL of affected individuals with hyperpigmentation is well demonstrated. Pigmentary disorders have been recognized to have a detrimental effect on the QoL of patients. Patients with skin conditions that cause hyperpigmentation, such as melasma and PIH commonly experience embarrassment, frustration, anxiety, and depression [20]. Taylor et al. studied the impact of pigmentary disorders on the QoL, and found that pigmentary abnormalities can cause feelings of embarrassment and self-consciousness. Many patients reported feeling unattractive and a need to try to cover up their skin. Patients also reported feeling that others focus on their skin rather than on what they are saying or doing and that their skin often affects their social and leisure activities [21]. Despite this, these conditions are frequently dismissed as benign or simply a cosmetic concern while other skin conditions such as psoriasis are not [22]. These disorders typically affect the face, which is harder to conceal than other parts of the body, further aggravating the patients concerns. Dermatologists should be aware of the physiological impact of these conditions and should screen the patients that need psychological follow up and encourage them to obtain additional support. Hyperpigmentation has a significant effect on self-image and self-esteem, compelling those afflicted to seek out dermatologic care in the form of prescription products. Despite these conditions not being debilitating or life-threatening, diagnoses of hyperpigmentation can cause deleterious emotional and psychological impact on affected individuals. Our findings describe a moderate effect on QoL especially in the domains of embarrassment and interference in daily activity. Interestingly, it has been reported that the DLQI of melasma is lower than vitiligo, lichen planus, bullous pemphigoid, acne scarring, and pityriasis rosacea [23]. This highlights the impact that hyperpigmentation disorders have on those affected with these conditions. The psychosocial aspect of hyperpigmentation has important implications for optimal management of patients. Special considerations when evaluating individuals with skin of color with facial hyperpigmentation can improve both cutaneous disease and QoL [24]. Given that disorders of hyperpigmentation are highly prevalent and affect a large portion of the patient population in dermatology, it is essential that dermatologists recognize the treatment gaps that exist in patients with skin of color as well as the importance of individualized treatment. By addressing these deficits, patients will receive optimal therapy, translating into improvements in results, satisfaction, and QoL.

## 5. Conclusions

Our findings highlight the negative impact that hyperpigmentation disorders can have on those affected, patients tend to feel embarrassed and this feeling interferes with activities of their daily lives. This demonstrates that pigmentary disorders are not merely a cosmetic concern and therefore, when examining the effects that hyperpigmentation disorders have on QoL, equal importance and a similar sensitivity of the psychosocial impact and disease burden should be given. Since the DLQI considers physical symptoms that are not relevant to pigmentary disorders, there may be a need for increased usage of specific scales designed specifically for hyperpigmentation disorders in order to truly capture the effects that these conditions have on patients [25]. Our study confirms that HQ remains the most prescribed bleaching agent, followed by TCC. However, patients felt that TCC provided the best result. The treatment of hyperpigmentation disorders is often difficult and prolonged, which leads patients to seek help from skin care specialists. Patients appear to trust and look for guidance in their dermatologist. This guidance points to positive trends, such as patients also using more sun protection in addition to prescription products, which is an essential part of any therapeutic regimen. Thus, it is the dermatologist’s duty to gauge the effect of the pigmentation disease on patients’ life in order to counsel, tailor, and decide on the most appropriate treatment option. Further research is needed to obtain a better understanding of hyperpigmentation disorders in skin of color and to develop new, improved, and safer therapies.

## Figures and Tables

**Table 1:** Comparison of characteristics between patients using or not using prescription lightening creams.

Characteristics *n* = 404 *	Prescription YES (*n* = 188, 46%)	Prescription NO (*n* = 216, 54%)	*p*-Value
Age (mean ± standard deviation)	41.3 ± 11.5	41.2 ± 13.1	0.923
Female	169 (89.9%)	189 (87.5%)	0.449
Skin Type I–III	57 (30.3%)	87 (40.3%)	0.037
Skin Type IV–VI	131 (70.7%)	129 (59.7%)	
Born in USA	52 (27.7%)	68 (31.5%)	0.401
Primary Language (*n* = 397)			0.065
English	75 (40.3%)	93 (44.1%)	
Spanish	73 (39.2%)	85 (40.3%)	
Portuguese	22 (11.8%)	9 (4.3%)	
Other	16 (8.6%)	24 (11.4%)	
Employed (*n* = 384)	132 (73.7%)	90 (45.7%)	0.502
Marital Status (*n* = 396)			0.687
Single	91 (49.5%)	107 (50.5%)	
Married	65 (35.3%)	81 (38.2%)	
Divorced	11 (6.0%)	7 (3.3%)	
Others	17 (10.2%)	17 (8.1%)	
Education (*n* = 395)			0.454
Less than college education	106 (57.3%)	113 (53.7%)	
College education	41 (22.2%)	62 (29.5%)	
Graduate school	38 (20.5%)	35 (16.7%)	
Hyperpigmentation Diagnosis			<0.001
Melasma	113 (60.8%)	61 (28.6%)	
Post-inflammatory hyperpigmentation	51 (27.4%)	80 (37.6%)	
Others	29 (11.8%)	72 (33.8%)	
Location of spots (*n* = 399)			0.008
Face	137 (74.9%)	129 (60.6%)	
Body	20 (10.9%)	42 (19.7%)	
Other	26 (14.2%)	42 (19.7%)	
Currently using a sunscreen (*n* = 397)			<0.001
Yes	144 (78.3%)	124 (58.2%)	
No	37 (20.1%)	87 (40.8%)	
Do not know	3 (1.6%)	2 (0.9%)	
Familiar with the term melasma (*n* = 396)			<0.001
Yes	65 (35.3%)	40 (18.9%)	
No	119 (64.7%)	172 (81.1%)	
Familiar with the term post-inflammatory hyperpigmentation (*n* = 393)			0.034
Yes	62 (34.3%)	52 (24.5%)	
No	119 (65.7%)	160 (75.5%)	
First go for help			
Internet	16 (8.5%)	36 (16.7%)	0.014
Friend	14 (7.4%)	28 (13%)	0.069
Doctor	139 (73.9%)	120 (55.6%)	<0.001
Family	17 (9.0%)	25 (11.6%)	0.405
Other	5 (2.7%)	9 (4.2%)	0.408
Best to treat spots (*n* = 398)			0.371
Primary care	3 (1.6%)	1 (0.5%)	
Dermatologist	178 (95.7%)	209 (98.6%)	
Aesthetician	1 (0.5%)	0 (0.0%)	
Friend with same condition	1 (0.5%)	0 (0.0%)	
Other	3 (1.6%)	2 (0.9%)	
Consult a doctor for prescription (*n* = 206)			<0.001
Yes	170 (93.4%)	0 (0.0%)	
No	8 (4.4%)	19 (79.2%)	
Do not know	4 (2.2%)	5 (20.8%)	
Product contain Hydroquinone (*n* = 393)			<0.001
Yes	117 (62.9%)	44 (21.3%)	
No	23 (12.4%)	109 (52.7%)	
Do not know	46 (24.7%)	54 (26.1%)	
Hydroquinone help lighten spots (*n* = 168)			0.956
Yes	43 (36.1%)	17 (34.7%)	
No	54 (45.4%)	22 (44.9%)	
Do not know	22 (18.5%)	10 (20.4%)	
Product contain azelaic acid (*n*= 380)			<0.001
Yes	11 (6.4%)	1 (0.5%)	
No	93 (54.4%)	144 (68.9%)	
Do not know	67 (39.2%)	64 (30.6%)	
Azelaic acid help lighten spots (*n*= 16)			0.769
Yes	2 (16.7%)	0 (0.0%)	
No	5 (41.7%)	3 (75.0%)	
Do not know	5 (41.7%)	1 (25.0%)	
Product called Tri-luma^®^ (*n*= 380)			<0.001
Yes	54 (31.4%)	1 (0.5%)	
No	58 (33.7%)	145 (69.7%)	
Do not know	60 (34.9%)	62 (29.8%)	
Tri-luma help lighten spots (*n* = 44)			0.090
Yes	30 (69.8%)	0 (0.0%)	
No	10 (23.3%)	0 (0.0%)	
Do not know	3 (7.0%)	1 (100.0%)	
Product contain Steroid (*n* = 375)			<0.001
Yes	17 (10.1%)	5 (2.4%)	
No	92 (54.8%)	149 (72.0%)	
Do not know	59 (35.1%)	53 (25.6%)	
Steroid help lighten spots (*n*= 20)			1.000
Yes	3 (23.1%)	1 (14.3%)	
No	5 (38.5%)	3 (42.9%)	
Do not know	5 (38.5%)	3 (42.9%)	
Total ^DLQI SCORE	7.59 ± 4.91	7.08 ± 5.46	0.330

* Denominators might differ due to missing data; ^DLQI = Dermatology Life Quality Index.

## Data Availability

The data presented in this study are available on request from the corresponding author.
